# Diagnostic accuracy of intrathecal kappa free light chains compared with OCBs in MS

**DOI:** 10.1212/NXI.0000000000000775

**Published:** 2020-06-11

**Authors:** Frida Duell, Björn Evertsson, Faiez Al Nimer, Åsa Sandin, Daniel Olsson, Tomas Olsson, Mohsen Khademi, Max Albert Hietala, Fredrik Piehl, Magnus Hansson

**Affiliations:** From the Department of Neurobiology, Care Sciences and Society (F.D.), Department of Clinical Neuroscience (B.E., F.A.N., T.O., M.K., M.A.H., F.P.), and Department of Laboratory Medicine (M.H.), Karolinska Institutet; Department of Clinical Chemistry (F.D., A.S., M.H.) and Department of Neurology (B.E., T.O., M.K., M.A.H., F.P.), Karolinska University Hospital; and Unit of Medical Statistics (D.O.), Department of Learning, Informatics, Management and Ethics (LIME), Karolinska Institutet, Stockholm, Sweden.

## Abstract

**Objective:**

To determine what kappa free light chain (KFLC) metric has the highest capacity to separate healthy patients from patients with MS, we evaluated the sensitivity, specificity, and the overall diagnostic accuracy of 4 different KFLC metrics. To assess the usefulness of KFLC in the diagnostics of MS, we compared the different KFLC metrics with oligoclonal bands (OCBs), the current gold standard biochemical method to demonstrate intrathecal antibody production.

**Methods:**

CSF and plasma were collected from patients with confirmed or suspected MS, other neurological diseases, as well as symptomatic and healthy controls between May 2017 and May 2018 (n = 335) at the Department of Neurology, Karolinska University Hospital, as part of routine diagnostic workup. KFLC analysis and isoelectric focusing for the detection of oligoclonal bands (OCB) were determined and correlated with diagnosis. Receiver operating characteristic (ROC) curve analysis was used to determine accuracy.

**Results:**

OCBs yielded a sensitivity of 87% and a specificity of 100%. All KFLC metrics showed a high sensitivity (89%–95%) and specificity (95%–100%). Using the optimal cutoff according to the Youden Index resulted for the KFLC intrathecal fraction in a cutoff of −0.41 with a sensitivity of 95% and a specificity of 97% and for CSF KFLC/CSF albumin with a cutoff of 1.93 × 10^−3^ with a sensitivity of 94% and specificity of 100%.

**Conclusion:**

All evaluated KFLC metrics have excellent accuracy, and both KFLC intrathecal fraction and CSF KFLC/CSF albumin are at least as good as OCB in separating patients with MS from a control group.

**Classification of evidence:**

This study provides Class III evidence that CSF KFLC accurately distinguishes patients with MS from healthy controls.

MS is a chronic neuroinflammatory disease where the inflammatory process comprises both cellular and humoral immune components. With 2.5 million people estimated to live with MS globally, it is one of the most common diseases of the nervous system. According to the most recent 2017 revision of the McDonald criteria,^1^ oligoclonal bands (OCBs) can substitute for dissemination in time, which previously required either another clinical relapse or support by MRI findings, thereby contributing to shortening diagnostic lag times. Especially in patients presenting with a first single clinical episode consistent with MS (clinically isolated syndrome [CIS]), an earlier diagnosis of MS is advantageous because early start of disease modulatory treatment is important to slow down further progression of disability and cognitive impairment.^2,3^ Selective OCB in CSF by isoelectric focusing (IEF), alongside an elevated IgG index, is the current gold standard biochemical method to demonstrate intrathecal antibody production. However, inherent characteristics of IEF make the procedure difficult to standardize and therefore prone to be affected by methodological factors such as gel quality, assessor bias, or presence of M-components. Alternative technical approaches circumventing these caveats without a pronounced loss of sensitivity or specificity are therefore warranted.

The fact that kappa free light chains in CSF (CSF KFLC) are increased in patients with MS has been known since 1974,^4^ and automated immunoassays for measurement of free light chains (FLCs) have been available for almost 2 decades. There is a growing body of evidence suggesting that determination of CSF KFLC is a valuable quantitative alternative or complement to the qualitative assessment of OCB.^5–16^ But KFLC can be presented in many different ways, as the pure CSF concentration or in more complex metrics where the permeability of the blood-brain barrier and the different kinetics of the molecules passing that barrier is taken into account. There is currently no consensus as to which metric to be used in a clinical setting. The hypothesis is that a more complex metric taking albumin index and other parameters into account will have a higher diagnostic accuracy than the pure CSF concentration of KFLC and that the diagnostic accuracy of KFLC will be comparable to OCB in the diagnosis of MS.

In this context, the primary objective of the current study is to define the KFLC metric with the highest diagnostic accuracy for MS; the second objective is to compare the diagnostic accuracy of KFLC and OCB for the same diagnosis.

## Methods

### Study population

All patients attending the Department of Neurology, Karolinska University Hospital, Sweden, between May 2017 and May 2018, where the analysis of KFLC in CSF had been performed (n = 410), were included. This also included some patients from the neurologic inpatient care. From this cohort, duplicates (n = 39), patients lacking a final diagnosis (n = 33), and patients where no plasma sample was available (n = 3) were excluded, resulting in the final study cohort (n = 335, table 1). Laboratory data were retrospectively collected from the laboratory's central electronic database. All CSF and blood samples were handled according to the consensus guidelines.^17^ Samples derived from the same sampling occasion were used for all analyses, i.e., when CSF was sampled, different aliquots were collected fresh and sent simultaneously to the laboratory for analysis. A clinical follow-up where the final diagnosis was set was performed on all patients after the time of testing. Chart review of all patients' medical history was performed by 2 MS-specialized neurologists. The chart review included initial and follow-up visits to identify and collect information about demographics, disease, and clinical characteristics at the time of sampling (table 1). The cohort was categorized according to the definitions and names proposed by the BioMS-eu consortium,^18^ and the diagnosis of relapsing-remitting MS (RRMS) was revised according both to the 2017 and the 2010 revision of the McDonald criteria.^1,19^ The 2017 revision was chosen in the definition of the whole cohort. This resulted in 7 subgroups: healthy controls (HCs), symptomatic controls (SCs), noninflammatory neurologic disease controls (NINDCs), peripheral inflammatory neurologic disease controls (PINDCs), central inflammatory neurologic disease controls (CINDCs), CIS or radiologically isolated syndrome, and MS (table e-1, links.lww.com/NXI/A259, displays a more in-depth definition of all patient groups). The control group was defined as HC and SC combined. When comparing OCB and KFLC, a subcohort of patients with MS was defined to ensure that the MS diagnosis was set based only on clinical and/or radiologic criteria for dissemination in space and time. That resulted in a control group (n = 60) and a group with RRMS (n = 62) defined by the revised McDonald criteria from 2010, hereinafter referred to as the STARD cohort. Brain MRIs, and when judged clinically relevant also MRIs of the spinal cord, were performed with 1.5 or 3 T scanners in all but a few patients. The scanning protocol included, but was not limited to, T2- and T1 sequences with and without contrast, fluid attenuation inversion recovery, and diffusion-weighted sequences. All images were read independently by 2 neuroradiologists.

**Table 1:**
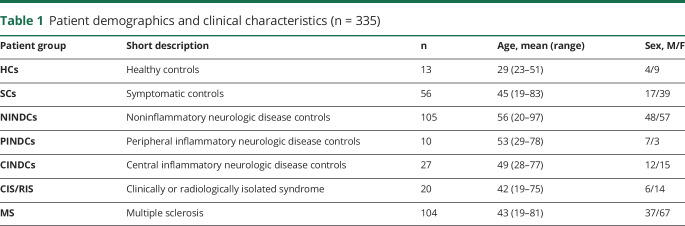
Patient demographics and clinical characteristics (n = 335)

### The index test

Paired CSF and plasma nephelometric measurements of KFLC were generated using a latex-enhanced immunonephelometry using a Dade Behring BNII nephelometric system with the N Latex FLC kappa reagent kit (Siemens, Marburg, Germany). The results are based on the proportion of light scatter from an antigen-antibody interaction. The lower detection limit was 0.034 mg/L. The reference interval of KFLC in plasma was 6.7–22.4 mg/L and in CSF was <0.34 mg/L according to the kit insert provided by the manufacturer. In the cases where multiple analyses of KFLC had been made on the same patient, the earliest KFLC result was chosen.

### The reference standard

OCBs were analyzed in 280 patients in 1 of 2 laboratories. A majority of the samples (n = 232) were analyzed in the in-house laboratory of the Neurology Department, Karolinska University Hospital, with IEF.^20^ Briefly, levels of IgG in plasma and CSF were quantified before the IEF to dilute the CSF and plasma samples equally. When the electrophoresis had been performed, the OCBs (if any) were visualized by immunoblotting in 3 steps. First, rabbit anti-human IgG Fc (Cat No IgG Q0331; Agilent, Santa Clara, CA) was added, followed by incubation with a biotinylated goat anti-rabbit IgG antibody (Cat No 65-6140; Thermo Fisher, Waltham, MA). Last, avidin-biotin-peroxidase complex was added with peroxidase staining with 3-amino-9-ethylcarbazole (Cat No 2122-10; BioVision, Milpitas, CA) as a substrate. The remaining samples (n = 48) were analyzed at the Karolinska University Laboratory with Hydragel CSF isofocusing (Sebia, Evry, France). In line with the McDonald criteria from 2017,^1^ 0–1 selective OCB was considered OCB negative, and 2 or more selective OCBs were considered OCB positive.^1^ For the remaining 55 samples in the cohort, no OCB assessment was requested and therefore not further investigated. Clinical information and index test result were available to the assessor of the reference standard. The same CSF and plasma samples were used for the analysis of the index test and the reference standard.

### Other measurements

Quantitation of albumin in CSF (CSF Alb) and plasma (P Alb) was performed using standard operating procedures using nephelometry (Dade Behring BNII; Siemens) and immunoturbidimetry (Cobas 8000 c701; Roche Diagnostics, Risch-Rotkreuz, Switzerland), respectively.

### Statistical analysis

The descriptive statistics and the ROC curve analyses were performed using GraphPad Prism 5® (GraphPad Software Inc., San Diego, CA) and Microsoft Excel®. Optimal cutoffs were estimated using the Youden Index, prioritizing sensitivity and specificity equally. The Youden Index was chosen because it is one of the most commonly used statistics used for summarizing the performance of a diagnostic test. The classification of evidence assigned to the primary research question is based on the information from the review.

### Standard protocol approvals, registrations, and patient consents

The use of clinical and laboratory data was approved by the Regional Ethical Review Board in Stockholm (Diary number: 2014/1201-31-1 and 2009/2107-31-2, respectively), but also included another study involving HC (Diary number: 2010/879-31-1). All study participants provided written informed consent.

### Data availability

Anonymized data will be shared by request from any qualified investigator.

## Results

**Figure 1:**
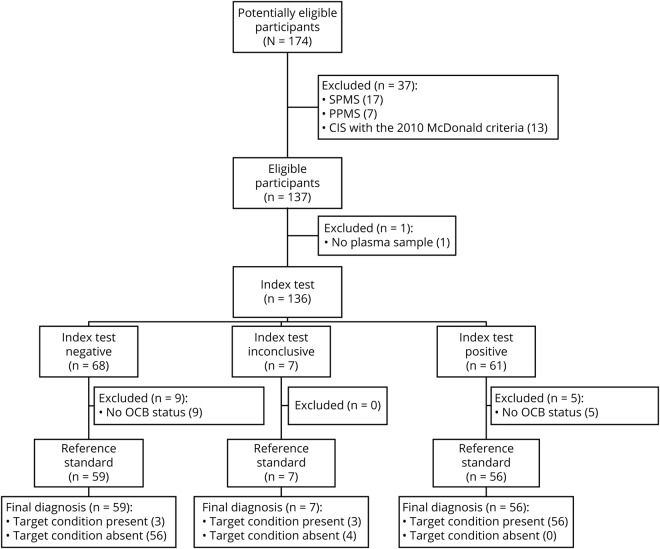
STARD flow diagram of index test kappa free light chain intrathecal fraction (KFLC IF) and reference standard OCB resulting in a control group (n = 60) compared with patients with MS (n = 62) KFLC IF below −0.5 = negative index test, KFLC IF −0/5 to 0 = inconclusive index test, KFLC IF above 0 = positive index test. n = 122 including inconclusive index test. CIS = clinically isolated syndrome; OCB = oligoclonal band; PPMS = primary progressive MS; SPMS = secondary progressive MS.

Patient demographics and clinical characteristics for the study cohort (n = 335) are shown in table 1. The HC group consisted of a younger population compared with the NINDC, PINDC, and CINDC groups, and the NINDC group also differed in age from the MS group. All groups except the PINDC group comprised a larger proportion of females, but the sex distribution across groups did not show any notable difference. Standards for Reporting of Diagnostic Accuracy Studies (STARD) protocol is presented in figure 1.

### KFLC concentrations and indices

**Figure 2:**
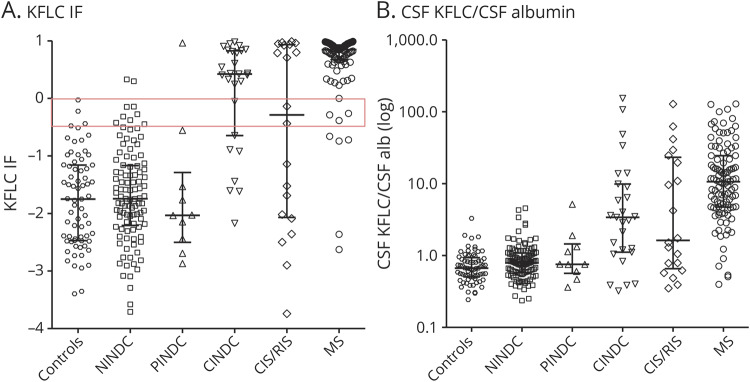
Distribution of KFLC in 6 different patient subgroups in the whole cohort (n = 335) (A) KFLC IF. Scatterplots show (A) KFLC IF with the gray zone (−0.5 to 0) marked with a red square and (B) CSF KFLC/CSF albumin for 6 different patient subgroups in the whole cohort (n = 335). Bars show medians and interquartile ranges. CINDC = central inflammatory neurologic disease control; CIS = clinically isolated syndrome; IF = intrathecal fraction; KFLC = kappa free light chain; NINDC = noninflammatory neurologic disease control; PINDC = peripheral inflammatory neurologic disease control; RIS = radiologically isolated syndrome.

Medians of KFLC metrics, albumin quotient (Q Alb), and OCB in the STARD cohort are shown in table 2. The distribution of KFLC intrathecal fraction (KFLC IF, for the definition of KFLC IF, see table e-2, links.lww.com/NXI/A259) and CSF KFLC/CSF Alb in the 6 different patient subgroups in the whole cohort is shown in figure 2. There was a big overlap in plasma KFLC (P KFLC) and P Alb between the control group and the patients with MS.

**Table 2:**
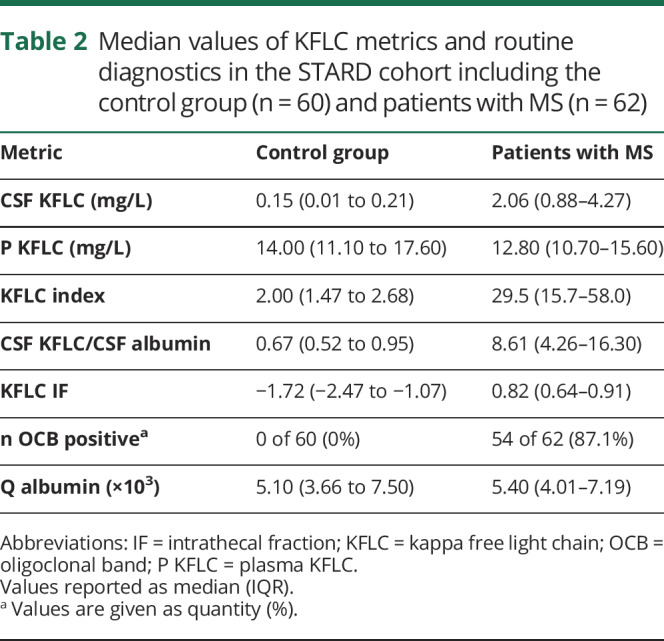
Median values of KFLC metrics and routine diagnostics in the STARD cohort including the control group (n = 60) and patients with MS (n = 62)

### Age and sex

When dividing our material into different age groups, a notable difference was observed in median interquartile range with higher CSF Alb median concentrations in men for all age groups (figure e-1, links.lww.com/NXI/A259). No considerable sex- or age-related differences were found for CSF KFLC (data not shown).

### Diagnostic performance

**Figure 3:**
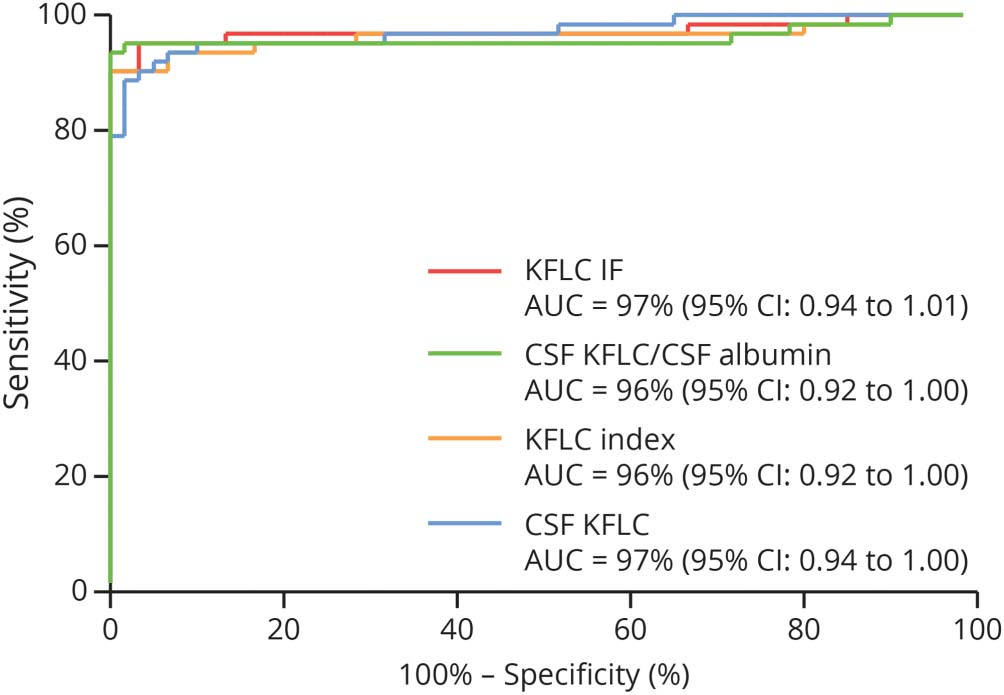
ROC curves of 4 different KFLC metrics in the STARD cohort ROC curves for KFLC IF, KFLC index, CSF KFLC, CSF KFLC/CSF albumin, and oligoclonal band (OCB) from samples in the prospective cohort with values for all metrics including OCB. ROC analysis performed for the control group (n = 60) vs patients with MS (n = 62). AUC reported as percentage with 95% CIs in parentheses. AUC = area under the curve; IF = intrathecal fraction; KFLC = kappa free light chain; ROC = receiver operating characteristic.

The AUC for KFLC IF, CSF KFLC/CSF Alb, KFLC index, and CSF KFLC was 97%, 96%, 96%, and 97%, respectively (figure 3). OCB yielded a sensitivity of 87% (54 of 62) and a specificity of 100% (0 of 60) (tables 2 and 3). Using the Youden Index, all KFLC metrics showed a higher sensitivity and a comparable specificity compared with OCB (table 3). The optimal cutoff for KFLC IF was −0.41 for a sensitivity of 95% and a specificity of 97%, and for CSF KFLC/CSF Alb, the optimal cutoff was 1.93 × 10^−3^ for a sensitivity of 94% and a specificity of 100%.

**Table 3:**
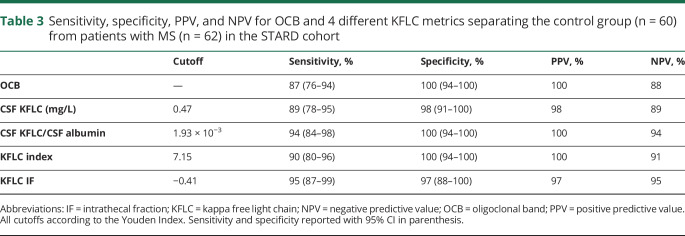
Sensitivity, specificity, PPV, and NPV for OCB and 4 different KFLC metrics separating the control group (n = 60) from patients with MS (n = 62) in the STARD cohort

### Elevated Q Alb

Elevated Q Alb serves as a proxy for blood-brain barrier damage. When looking only at the samples with an elevated Q Alb, control group (n = 14), and patients with MS (n = 16), the AUCs of the ROC curves for KFLC IF, CSF KFLC/CSF Alb, KFLC index, and CSF KFLC were all 100% (data not shown). Using the Youden Index cutoffs in this cohort gave 2 false-negative tests with OCB and KFLC index and 1 false-positive test with CSF KFLC. KFLC IF and CSF KFLC/CSF Alb could fully separate the 2 groups (table 4).

**Table 4:**
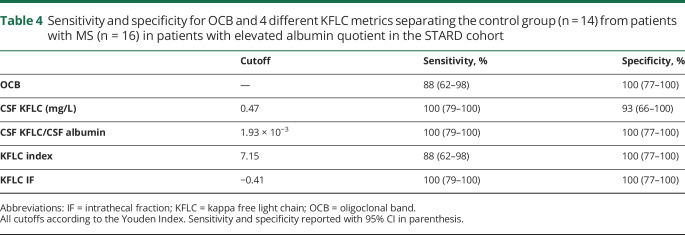
Sensitivity and specificity for OCB and 4 different KFLC metrics separating the control group (n = 14) from patients with MS (n = 16) in patients with elevated albumin quotient in the STARD cohort

### Discrepant results

Discrepant results for OCB and KFLC IF were present in 5 patients with MS, all of whom where OCB negative. Three of them had KFLC IF > 0, and 2 had KFLC IF > −0.41. There was a tendency toward milder MRI lesions (smaller and fewer lesions).

## Discussion

The primary objective of this study was to define the KFLC metric with the highest diagnostic accuracy for MS. Because most of the previously conducted studies have been retrospective involving analysis of archival samples in non–real-time clinical conditions, the aim was to do this evaluation in a prospective setting. Our main finding is that KFLC IF, CSF KFLC/CSF Alb, KFLC index, and CSF KFLC all have a higher sensitivity compared with OCB in discriminating patients with MS from the control group without pronounced, if any, loss of specificity. The 2 metrics showing the highest sensitivity are KFLC IF and CSF KFLC/CSF Alb with 95% and 94%, respectively.

According to Presslauer et al.,^6^ KFLC IF with 0 as a cutoff is the most efficient metric to separate patients with MS from controls. In a pilot study, which preceded this study (n = 75, data not shown), the optimal cutoff for KFLC IF was found to be −0.5. For this reason, a cutoff of 0 with a gray zone from −0.5 to 0 was used. The optimal cutoff for KFLC IF in this cohort was −0.41, which is well within our previously established gray zone. A cutoff for a KFLC metric giving priority to sensitivity over specificity would result in clinicians being able to establish signs of intrathecal immunoglobulin production in a larger fraction of patients fulfilling clinical and neuroradiologic criteria of MS. However, this would affect negatively on specificity and the risk of false-positive result. Therefore, it seems sensible to use a gray zone.

One theoretical advantage of KFLC IF over CSF KFLC/CSF Alb is that it considers the difference in molecular sizes between the free kappa chain and albumin using a nonlinear relation of KFLC influx into the CSF relative to the Q Alb.^21^ The advantages of CSF KFLC/CSF Alb are that the cost is reduced by half compared with KFLC IF and that no serum sample is needed. However, a disadvantage is that this metric will not correct for spontaneous methodological variations, a problem that is reduced in metrics where the same method is used to analyze parameters found in both the nominator and the denominator (such as KFLC IF and KFLC index).

Of interest, while largely overlapping, there was a small fraction of patients with OCB-negative MS with elevated or gray zone concentrations of KFLC IF. The relatively mild MRI lesions in these patients could indicate a low disease burden. We therefore hypothesize that the discrepancy could represent a higher sensitivity of KFLC IF compared with OCB. Additional studies are needed to establish the immunologic basis for this discrepancy including continuously sampled CSF.

A sex difference in Q Alb has recently been demonstrated.^22^ We confirm that CSF Alb is affected not only by age but also by sex, with men in general having higher levels of CSF Alb compared with females. In the subcohort with elevated Q Alb, OCB and KFLC index resulted in 2 false-negative tests each and CSF KFLC resulted in 1 false-positive test. Although this subcohort is too small to draw any conclusions from, these findings along with the confirmed variations of Q Alb due to age and sex stress the importance of including a parameter controlling for blood-brain barrier leakage in the equation.

A limitation of the study was that a small proportion of the patients lacked OCB status. Also, the sizes of the non-MS populations were relatively small, making conclusions in these patient groups less reliable. Another limitation was that the results are obtained from a single laboratory, and the generalizability of the cutoff values is limited to this setting, making it necessary to adjust for the performance of local analytical equipment. Also, it is important to note that intrathecal production of KFLC is a nonspecific marker of neuroinflammation or B-cell activity, similar to the intrathecal presence of IgG or a positive OCB status. Diagnostic sensitivity and specificity of KFLC in patients with MS are therefore expected to decrease when comparisons are made across different inflammatory neurologic diseases (figure 2). Furthermore, kinetics of CSF KFLC are not known in detail. For example, reduced glomerular filtration rate, dialysis, presence of an M-component of light chains, or multiple myeloma may in theory affect the level of P KFLC, and as a consequence also the CSF KFLC concentration, which may have an impact on the interpretation of the KFLC metrics. Despite this, the fact that a higher proportion of patients fulfilling clinical MS criteria were positive for intrathecal immunoglobulin production with KFLC than OCB is highly encouraging. Importantly, in contrast to OCB, KFLC also has the capacity to detect IgM-producing cell clones common in early inflammatory responses and possibly also having a prognostic significance in MS.^23,24^ A further advantage is the possibility to more reliably quantify intrathecal immunoglobulin production, which may be relevant when monitoring certain disease-modulating treatments, in particular those involving depletion of B-cell subsets. Finally, KFLC is faster and more cost effective than standard techniques used to determine OCB status.

To answer our first objective of what KFLC metric has the highest diagnostic accuracy for MS, we found that all 4 evaluated KFLC metrics had excellent accuracies with AUC of ROC curves above 95%. The 2 metrics showing the highest sensitivities without considerable, if any, loss in specificity were KFLC IF (sensitivity 95% and specificity 97%) and CSF KFLC/CSF Alb (sensitivity 94% and specificity 100%).

To answer our second objective regarding the comparison of OCB and the KFLC metrics, we found that all 4 evaluated KFLC metrics had equal or higher sensitivities compared with OCB in discriminating patients with MS from the control group without considerable, if any, loss of specificity.

Compared with OCB, KFLC has the added advantages of being objective, easier to standardize, faster, and less costly. We therefore suggest KFLC IF or CSF KFLC/CSF Alb as a valid substitute for OCB in the diagnostics of MS. When choosing what KFLC metric to use in the clinical setting, one must take methodological, economical, and to some extent also theoretical aspects into account. Additional studies are needed to provide the comparative performance of KFLC in other inflammatory and noninflammatory diseases.

## Glossary

CINDC: central inflammatory neurologic disease control
CIS: clinically isolated syndrome
FLC: free light chain
HC: healthy control
IEF: isoelectric focusing
IF: intrathecal fraction
KFLC: kappa free light chain
NINDC: noninflammatory neurologic disease control
OCB: oligoclonal band
P Alb: plasma albumin
PINDC: peripheral inflammatory neurologic disease control
Q Alb: albumin quotient
SC: symptomatic control
